# Emerging concepts and spectrum of renal injury following Intravesical BCG for non-muscle invasive bladder cancer

**DOI:** 10.1186/s12894-017-0304-5

**Published:** 2017-12-06

**Authors:** Azharuddin Mohammed, Zubair Arastu

**Affiliations:** Royal Shrewsbury Hospital, Mytton Oak Road, Shrewsbury, SY3 8XQ UK

**Keywords:** Intravesical bacillus Calmette-Guerin, BCG renal complications, Interstitial nephritis, Granuloma, AKI, CKD, Nephrotic syndrome

## Abstract

**Background:**

Intravesical Bacilli Calmette-Guerin (IVBCG) therapy for non-muscle invasive bladder cancer (NMIBC) has long been in use successfully. Albeit rarely, we still face with its safety concerns more than 25 years on since its approval by US Food and Drug Agency in 1990. Local and systemic infection following intravesical BCG is widely reported as compared to immune mediated local or systemic hypersensitivity reactions involving kidneys; acute kidney injury (AKI) and other renal manifestations are well reported but not of chronic kidney disease (CKD).

**Case:**

An interesting case of a female was referred to nephrologists in advanced stages of CKD at an eGFR of 10 ml/min/1.73^2^ following IVBCG for NMIBC. Our patient’s renal function plateaued when IVBCG was held; and worsened again when reinstilled. It introduces the concept of ‘repetitive’ immune mediated renal injury presenting as progressive CKD rather than AKI, as is generally reported. Although response was poor, corticosteroids stopped CKD progression to end stage renal disease.

**Conclusions:**

We highlight the need for increased awareness and early recognition of IVBCG renal complications by both urologists and nephrologists in order to prevent progressive and irreversible renal damage. Low incidence of IVBCG renal complications may also be due to under recognition in the era prior to CKD Staging and AKI Network (and AKI e-alerts) that defined AKI as a rise in serum creatinine of ≥26umol/L; hence an unmet need for urgent prospective studies. Major literature review focuses on emerging spectrum of histopathological IVBCG related renal complications and their outcomes.

## Background

Intravesical Bacilli Calmette-Guerin (IVBCG) therapy for non-muscle invasive bladder cancer (NMIBC) has long been in use successfully since 1973. Albeit rarely, we are increasingly facing its renal complications more than 25 years on, since its approval by US Food and Drug Agency in 1990. Renal injury following IVBCG is thought to be due to ascending infection or rarely due to granulomatous interstitial nephritis presenting as acute kidney injury/acute renal failure (AKI/ARF)). Case reports of acute renal injuries such as glomerulonephritis (GN), nephrotic syndrome (NS), rhabdomyolysis and rapidly progressive glomerulonephritis (RPGN) are increasing including fatal consequences in some. However, reports of chronic kidney disease (CKD) are not published, which and may be due to under-recognition. We report here an interesting case of advanced CKD presenting in stage 5 following IVBCG with novel insights into pathological process and guiding management plans.

## Case

A 73 year-old Caucasian female referred to Nephrology with an eGFR of 10 ml/min/1.73^2^ in June 2016; it was 60 ml/min/1.73^2^ in Feb 2015. There was progressive decline of eGFR that dropped to 14 ml/min/1.73^2^ in March 2016. There was no history of weight loss, recurrent UTI, new medication use or any autoimmune/vasculitc symptoms. Past medical history included well-controlled hypertension for 4 years and gastro-oesophageal reflux. Medications included Lisinopril, Lacidipine and Omeprazole. In November 2014, she had resection of NMIBC staged as high-grade cT1NxMx and receiving scheduled IVBCG (Onco tice12.5 mg) instillations since April 2015. Her IVBCG therapy interrupted due to a national shortage; it recommenced and by the time of referral received 16 instillations until May 2016.

She looked well on examination with BP of 155/69 mmHg and systemic examination was unremarkable with no rash, tender nodules, red eyes or lymphadenopathy. Urine dipstick showed leukocytes ++, protein + and culture showed no growth. Serum creatinine was 357 umol/L (eGFR 10 ml/min/1.73^2^). Immunology came negative for ANA, ANCA, Anti GBM, myeloma screen, Hepatitis B and C and Complement levels C3/C4; ultrasound of the kidneys, ureter and bladder was normal. An ultrasound guided renal biopsy performed due to rapidly progressive unexplained decline in kidney function.

Renal biopsy showed interstitial inflammation with moderate lymphocytic infiltration, eosinophil’s and a non-caseating granuloma. Background chronic tubular atrophy and some acute changes noted but no tubular inflammatory infiltrate. Immunofluorescence was negative and no AFB demonstrated.

Histological features were suggestive of a ‘drug-like’ immune mediated hypersensitivity reaction secondary to IVBCG. This was further supported by- patient’s ethnicity, absence of previous TB exposure (and current symptoms), any new medications, normal CXR, serum ACE levels, negative Mantoux/TB Interferon assay and negative urine cultures for MTB.

As the last dose of IVBCG was within 6 weeks and acute on chronic biopsy picture, trial of steroids alone commenced (Prednisolone 40 mg daily); aim was to stop further decline in kidney function and buy time for dialysis preparation. It is now increasingly recognised that dialysis patient’s outcomes are better who see a nephrologist before dialysis initiation compared to those who don’t [1].

**Fig. 1:**
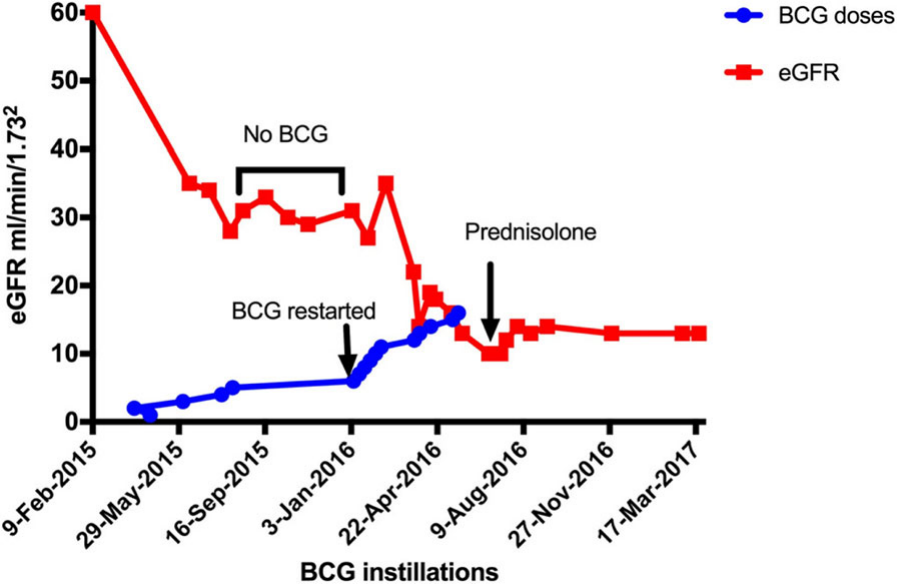
Graph illustrating effect of intravesical BCG on renal function during treatment and following discontinuation

Patient responded partially to 6 weeks of steroids; eGFR improved from 10 to 14 ml/min/1.73^2^ and then stabilised at 12–13 ml/min/1.73^2^ at 9-month follow-up obviating any need for dialysis (Fig. 1). There were no systemic features of BCG infection and repeat cystoscopy showed no recurrence of NMIBC.

## Discussion

Urothelial carcinoma is the eleventh most frequent cancer in women with mean age of 60 years and rising incidence in over 50 years [2]. NMIBC accounts for 75–90% of these with poor prognosis in females. Immunotherapy for NMIBC remains the gold standard with efficacy of IVBCG shown to be better than chemotherapy in reducing recurrence, progression and mortality [3]. The total cost for NMIBC is reported to be £32.25(2001–2002) million in UK and $3.7 billion in USA for 2001 [4].

### Use of Intravesical BCG in NMIBC

BCG is a live attenuated vaccine obtained from Mycobacterium bovis. It has been used since the first seminal paper of Morales et al. in 1976 [5, 6]. However, its use increased following FDA approval in 1990 providing more data on its safety and efficacy over the last quarter of a century.

#### Dose

Practice patterns vary but standard regimen of IVBCG for high-grade NMIBC is induction (6 consecutive weekly doses) with 1–3 years maintenance (3 consecutive weekly doses) at 3, 6, 12, 18, 24, 30, and 36 months.

#### Mechanism

Mechanism of action is unknown but local immune response following phagocytosis of BCG antigen is well recognised. Antitumor effect and cytotoxicity is mediated via cytokine release and soluble factors such as TNF- α, IFN- γ and NO [7].

#### Adverse effects

IVBCG is generally well tolerated albeit minor self-limiting adverse effects in 10–50% patients that includes flu-like symptoms, malaise and local bladder irritability causing dysuria, frequency and urgency. They occur 4–6 h after instillation and between 3 and 7 doses of IVBCG and do not require any treatment. However, fatality reports due to systemic reaction raised concerns on its established safety profile [8, 9]. Early IVBCG years witnessed haematogenous spread (lungs, liver, kidney, peritoneum, prostate and testis) after traumatic/non-traumatic catheterisation; consequent infection and DIC rarely caused multi organ failure and death [9–12]. Lately, increasing publications noticed on immune complications such as hypersensitivity, anaphylactoid purpura, Henoch Schönlein Purpura (HSP) and glomerular and tubulo-interstitial injuries [13, 14]. We exclusively review here renal complications of IVBCG.

Reported spectrum of renal injury following IVBCG since 1990 is listed above (Table 1) and the data summarised below:

- **Asymptomatic renal granuloma (** ***n*** **= 4):** Incidentally detected on follow-up imaging and seem to show favourable prognosis. Granuloma resolved with Anti-Tuberculous therapy (ATT) in one case and required nephro-ureterectomy in another due to left renal pelvic tumour. However, recent case series managed conservatively for 2 years showed no systemic features or progression [15, 16].
- **AKI due to interstitial nephritis with/without granuloma (** ***n*** **= 8):** This is the commonest presentation reported with elevated serum creatinine levels. Analysis of these cases highlights following:
  - ◦ Biopsies showed significant interstitial inflammation with moderate fibrosis; granulomas seen in only 50%.
  - ◦ Therapies involved prednisolone alone and pulsed methylprednisolone with/without ATT.
  - ◦ Outcome: Three (37.5%) patients were able to come off dialysis with above regimes; rest showed partial recovery.
- **AKI with Glomerulonephritis (** ***n*** **= 2):** In one case, mild renal dysfunction was associated with hypertension, haematuria and proteinuria. It responded well to steroids and ATT. Another case from Japan recently reported RPGN with fatal outcome in an elderly male [17]. Importance of history of recent IVBCG use was emphasised as well as fatal infection risk following immunosuppression and plasma exchange amongst elderly.
- **Nephrotic syndrome – Membranous nephropathy (** ***n*** **= 1):** This is the only case reported to date in adults. Although malignancy and infection associated nephrotic syndrome is well known, authors reported this case to be due to IVBCG. Remission achieved with prednisolone 80 mg daily and ACEi after a month, and maintained at 6 months [18].
- **AKI with Haemolytic uremic syndrome, Rhabdomyolysis and Multiorgan Failure (** ***n*** **= 1):** Patient developed multi organ failure after the eighth standard dose and died despite maximum intensive care management and plasma exchange. It calls for increased clinician awareness of rare but serious IVBCG complications.

**Table 1:** Clinical spectrum of renal presentations, treatment and their outcome following Intravesical BCG

Year/ Ref	Age	Sex	Clinical Presentation	Initial SCr(mg/dL)	BCG Strain	BCG Instillations	Renal Histology	Granuloma	Treatment	Recovery	Last SCr(mg/dL)
1991 [21]	70	M	VH, Raised Cr	3	Pasteur	11	Interstitial epitheloid granulomas	Yes	I + P	Partial	1.8
1991 [21]	70	M	ARF	5.4	Pasteur	18	Interstitial nephritis with mesangial IgM and C3 deposits	No	I + E + P	Poor Died	Off HD; CrCl 10
1991 [21]	48	M	Hematuria and Proteinuria	1.3	Pasteur	9	Diffuse mesangial proliferation with subendothetial deposits of IgG + C3 and moderate interstitial fibrosis;	No	I + R	Complete	
2000 [8]	72	M	ARF- HUS, Rhabdomyolysis	3.8	?	8	No biopsy as patient too unwell	N/A	Pl.Ex, HD	None Died	–
2000 [25]	67	M	UTI		?		Renal caseating granulomas	Yes	I + R + Pip	Complete	–
2001 [14]	57	F	UTI	–	Tokyo	5	–	NA	Pulse CS	Complete	–
2001 [14]	76	M	UTI	–	Tokyo	6	–	NA	Pulse CS	Complete	–
2005 [22]	72	M	ARF	2.9	Tice	9	Acute tubulointersttial nephritis. Mesangial proliferation + focal segmental changes;IF-ve	No	I + R + P	Partial	1.9
2006 [23]	72	F	ARF	3.1	Connaught	5	Diffuse Interstitial Nephritis +2 non-necrotising Granulomas; IF nonspecific IgM + C3	Yes	Pred alone	Complete	1.3
2007 [24]	76	M	ARF	6.5	?	10	Diffuse and severe interstitial nephritis	No	MP, Pred	Partial	3.4
2007 [18]	54	M	Nephrotic Syndrome	Normal	?	12	Membranous glomerulonephritis - IgM, C3 and IgG+	No	Pred alone	Complete Remission	Normal
2013 [12]	76	M	AKI	7.9	?	10	Tubulointerstitial nephritis with moderate eosinophilic infiltrate	No	Oral MP, ATT, HD	Partial	Off HD; 2.5
2015 [15]	52	M	Surveillance CT	1.2	Onco Tice	18	Necrotising granuloma with no interstitial inflammation; IF not done;	Yes	ATT	Complete	1.2
2015 [16]	68	M	VH	–	OncoTice	18	Non-necrotising granulomas	Yes	None	Complete	–
2015 [16]	74	M	NVH	–	OncoTice	9	Chronic Granulomatous Interstitial Nephritis	Yes	None	Complete	–
2017 [17]	80	M	AKI-RPGN HSP	3.6	Connaught	8	IgA-Fibrinoid necrosis + 20% crescents Mesangial IgA. Skin- HSP vasculitis	No	MP, Pred Pl.Ex, HD	Partial Died	Off HD; 2.8
Present	73	F	Advanced CKD5	4	OncoTice	16	Interstitial nephritis with granuloma and acute/chronic tubular damage, IF negative	Yes	Pred alone	Poor	3.3

*AAT* Antituberculous Therapy, *AKI* Acute Kidney injury, *ARF* Acute renal failure, *CS* Corticosteroids, *E* Ethambutol, *HD* Hemodialysis, *HSP* Henoch ≈Schönlein Purpura, *HUS* Haemolytic Uremic Syndrome, *IgA* Immunoglobulin A, *I* Isoniazid, *MP* Methyl Prednisolone, *NVH* Non-Visible Haematuria, *Pip* Piperacillin, *Pl.Ex* Plasma Exchange, *Pred* Prednisolone, *P* Pyrazinamide, *R* Rifampicin, *SCr* Serum creatinine, *VH* Visible Haematuria, *RPGN* Rapidly Progressive Glomerulonephritis, *UTI* Urinary Tract Infection

### Renal toxicity

#### Incidence

Reliable data on incidence of IVBCG renal toxicity is lacking due to various patient and treatment related factors discussed below. Under-recognition is probably significant prior to CKD Staging (using eGFR) and AKI Network era that defined AKI as a rise in serum creatinine of ≥26umol/L. A large study (*N* = 2602) by Lamm et al. in 1992 reported 2 cases (0.1%) of renal abscess, seen only with Connaught strain of BCG [19]. Manufacturers of one BCG strain stated 9% renal toxicity in their study arm (*N* = 112) with 1.8% in ≥ Grade 3 cancer [9].

#### Patient factors

This is the second case of renal failure in females, which could be due to gender differences of NMIBC, M: F ratio of 3:1. High environmental and industrial carcinogen exposure in men like aromatic amines in cigarette smoke, dye and rubber industry may also explain this difference.

#### Trauma, UTI and site

Traumatic catheterization and concurrent cystitis are well known factors for high-risk of serious adverse events and a close monitoring can reduce toxicity. Upper tract urothelial carcinoma and evidence of vesicoureteric reflux are other important factors to consider [20].

#### Strain type

Repeated culture and attenuation of Mycobacterium Bovis produces BCG strains of variable immunogenicity, virulence and toxicity, which limits obtaining absolute comparative toxicity data for all strains reliably. Renal abscess seen with Connaught strain [19]. Most BCG strains cause renal toxicity including Pasteur, Onco Tice, Connaught, and Tokyo strains [15, 16, 21–24].

#### Dose

Toxicity reported to be dose dependent in addition to strain specific. Analyses of cases show no correlation of severity of kidney injury to number of instillations with an exception of rare fatality after eight instillations [8] (Table 1). It becomes a hard clinical choice to limit BCG instillations when no reliable alternatives to radical cystectomy are available that reduce recurrence and progression of NMIBC and maintain functional urinary bladders.

#### Repetitive injury

Almost all renal injury cases reported AKI *during or after* administration of standard or higher doses. Interestingly, our case shows that there might be a window of opportunity when interrupting therapy could lead to stabilization of renal function. A national shortage of IVBCG interrupted patient’s therapy; renal function trend during this period demonstrated that eGFR plateaued. More intriguing was a decline in renal function again following readministration of IVBCG suggesting a cumulative dose related repetitive injury that could present as CKD rather than a one-hit process causing AKI (13, 14, 17–19)Fig 1. Therefore, early recognition of renal dysfunction during IVBCG treatment is vital.

#### Granuloma

**Fig. 2:**
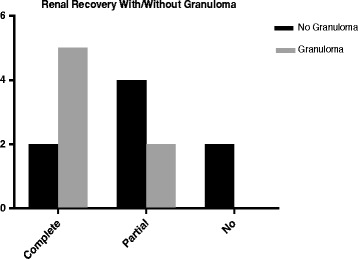
Renal outcome in Intravesical BCG related granulomatous and non-granulomatous renal disease

Presence of renal granuloma in the context of IVBCG requires comprehensive patient evaluation for infectious, autoimmune and non-infectious causes such as sarcoidosis and medications. Case series analysis (*n* = 15) show that although the median numbers of instillations in granulomatous cases were non-significantly higher, serum creatinine at presentation was lower and had no fatalities compared to non-granulomatous presentation (Table.2, Fig 2). This suggests complex immune-pathological mechanisms for renal injury that depends on strain virulence, toxicity and host response, which may already be low in this age group. However, as the data is limited formal conclusions cannot be drawn or generalised, highlighting need for urgent prospective studies.

**Table 2:** Demographics and clinical differences between Intravesical BCG related granulomatous and non-granulomatous disease

	No. Granuloma (*n* = 8)	Granuloma (*n* = 7)	p =
Gender n = (M, F)	8, 0	5, 2	NA
Age (Years)	72 (58–76)	70 (67–73)	0.48
No. of Instillations	9.5 (8.25–11.5)	13.5 (8–18)	0.40
BCG Strains	Pas, Tice, Conn	Pas, Onc Tice, Conn	
Peak creatinine (mg/dL)	4.6 (1.9–6.4)	3 (1.7–3.8)	0.28
Required HD (n=)	3	0	
Death (*n* = 3)			
17 -day	1		
32-day	1		
1-year	1		
Recovery (n = 15)			–
Partial/None	6	2	–
Complete	2	5

## Key messages

- ▪ Spectrum of renal injury following IVBCG can present:
  • After standard doses
  • Without any constitutional symptoms
  • Early or late stages (of AKI/CKD) requiring dialysis.
- ▪ Cumulative toxicity with repetitive doses needs consideration for preventing avoidable progressive renal damage.
- ▪ Renal biopsy even in late stages can guide management, prognosis and exclude malignancy in uncertain cases.
- ▪ Good response to corticosteroids can obviate dialysis dependence even in late stages; partial response can avert an unplanned dialysis as seen in our case.

## Conclusion

- ◦ Majority of patients do benefit from IVBCG; nevertheless, rarely it causes a spectrum of immune mediated renal injuries that include granulomatous/non-granulomatous interstitial nephritis, glomerulonephritis, nephrotic syndrome and systemic (and local) hypersensitivity reactions.
- ◦ A decline in kidney function, haematuria and proteinuria during BCG treatment, in the absence of identifiable causes, should prompt suspicion of treatment related renal complication and an early nephrology referral considered for further evaluation and consideration of renal biopsy.
- ◦ Risk of AKI with/without CKD (rarely advanced renal failure) would need to be included in the IVBCG Patient Information Leaflets (and Consent Forms) with a plan for scheduled renal function monitoring for occurrence of AKI during and up to 90 days after therapy as per standard practice.
- ◦ Urgent prospective observational studies can help assess the true incidence of IVBCG renal toxicity using the newer AKIN based AKI e-alerts and CKD Staging.

## Abbreviations

AKI: Acute Kidney injury
AKIN: Acute Kidney Injury Network
ARF: Acute Renal Failure
ATT: Antituberculous therapy
BCG: QBacilli Calmette-Guerin
CKD: Chronic Kidney Disease
CS: Corticosteroids
DIC: Disseminated Intravascular Coagulation
eGFR: estimated Glomerular Filtration Rate
HD: Hemodialysis
HSP: Henoch Schonlein Purpura
HSP: Henoch Schönlein Purpura
HUS: Hemolytic Uremic Syndrome
MP: Methyl Prednisolone
MTB: Mycobacterium Tuberculosis
NMIBC: Non Muscle invasive Bladder Cancer
NS: Nephrotic Syndrome
NVH: Non-Visible Hematuria
Pl.Ex: Plasma Exchange
RPGN: Rapidly Progressive Glomerulonephritis
UTI: Urinary tract Infection
VH: Visible Hematuria
